# Dissociating the semantic function of two neighbouring subregions in the left lateral anterior temporal lobe

**DOI:** 10.1016/j.neuropsychologia.2014.12.004

**Published:** 2015-09

**Authors:** Ana Sanjuán, Thomas M.H. Hope, 'Ōiwi Parker Jones, Susan Prejawa, Marion Oberhuber, Julie Guerin, Mohamed L. Seghier, David W. Green, Cathy J. Price

**Affiliations:** a Wellcome Trust Centre for Neuroimaging, University College London, London WC1N 3BG, United Kingdom; b Neuropsychology and Functional Imaging Group, Departamento de Psicología Básica, Clínica y Psicobiología, Universitat Jaume I, Castellón, Spain; c Wolfson College, University of Oxford, Oxford OX2 6UD, United Kingdom; d Experimental Psychology, University College London, London WC1E 6BT, United Kingdom

**Keywords:** fMRI, Semantic memory, Anterior temporal lobe, Naming

## Abstract

We used fMRI in 35 healthy participants to investigate how two neighbouring subregions in the lateral anterior temporal lobe (LATL) contribute to semantic matching and object naming. Four different levels of processing were considered: (A) recognition of the object concepts; (B) search for semantic associations related to object stimuli; (C) retrieval of semantic concepts of interest; and (D) retrieval of stimulus specific concepts as required for naming. During semantic association matching on picture stimuli or heard object names, we found that activation in both subregions was higher when the objects were semantically related (mug–kettle) than unrelated (car–teapot). This is consistent with both LATL subregions playing a role in (C), the successful retrieval of amodal semantic concepts. In addition, one subregion was more activated for object naming than matching semantically related objects, consistent with (D), the retrieval of a specific concept for naming. We discuss the implications of these novel findings for cognitive models of semantic processing and left anterior temporal lobe function.

## Highlights

• We examined the role of the lateral anterior temporal lobe (LATL) in semantic matching and naming.
• We found a different pattern of activation in two LATL sub-regions.
• Both subregions take part in the successful retrieval of amodal semantic concepts.
• One sub-region was more related to the retrieval of a specific concept for naming.

## 1 Introduction

The role of the anterior temporal lobe in semantic memory has been highlighted by studies of patients with semantic difficulties and functional imaging studies of healthy participants (for reviews, see Binder et al. (2009); Binder and Desai (2011); Lambon Ralph and Patterson (2008); Lambon Ralph (2013), Patterson et al. (2007); Price (2012); Wong and Gallate (2012)). Here, we focus on the functional responses in and around a left lateral anterior temporal lobe (LATL) region that was localised by Pobric et al. (2010) as a site where semantic matching performance was impaired during a temporary “virtual lesion” produced by repetitive transcranial magnetic stimulation (rTMS). The location of the TMS testing site was based on previous studies that reported the contribution of the same LATL region to synonym judgements and naming tasks on written words and pictures (Binney et al., 2010; Pobric et al., 2007; Lambon Ralph et al., 2009) using non-semantic control conditions such as number judgements. More recently, Visser et al. (2012) used the same semantic matching paradigm as Pobric et al. (2010) in an fMRI study and found activation in the same LATL region for the semantic compared to a perceptual matching task.

**Fig. 1:**
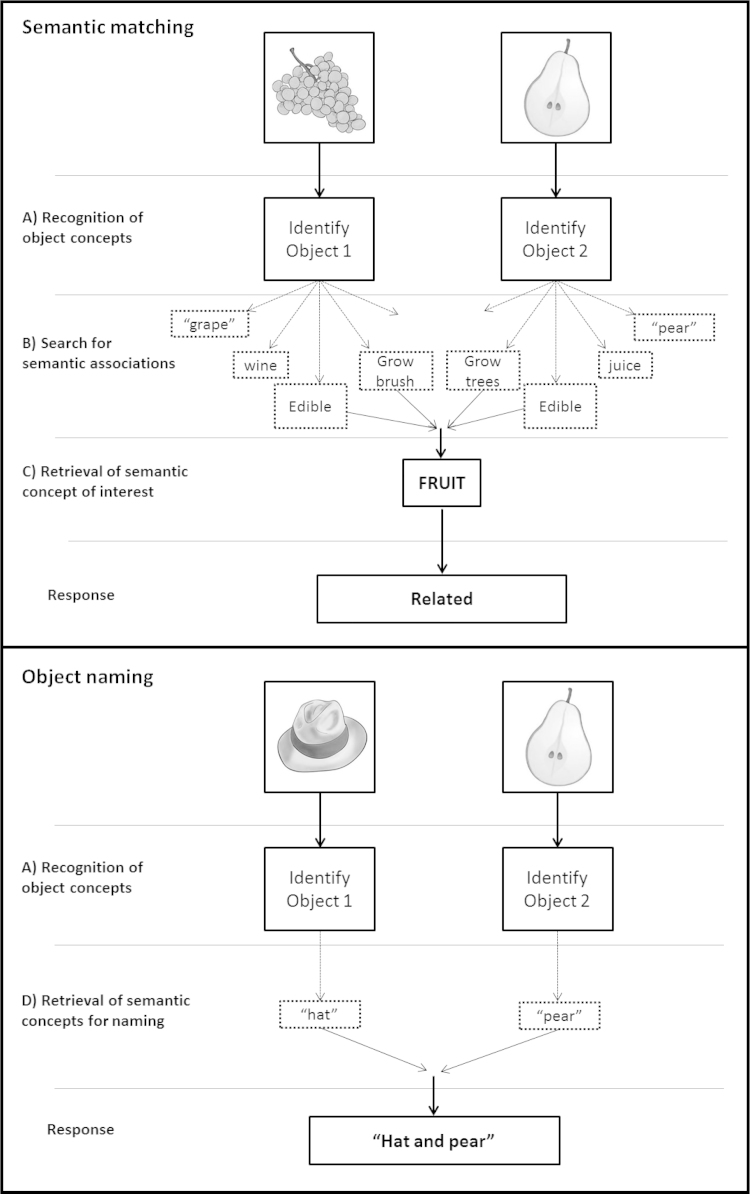
Task analysis for a single semantic matching and object naming trial. Top panel: Semantic matching trial: Deciding whether two stimuli are related or unrelated, involves (A) recognising object concepts; (B) searching for associations related to each object concept; and C) retrieving a specific semantic concept for matching. Bottom panel: Object Naming trial: Naming a concept involves(A) recognising object concepts; and (D) retrieving a specific concept for naming.

In both Pobric et al. (2010) and Visser et al. ( 2012), the semantic task of interest consisted of semantic association decisions on pictures of objects or their written names, using a paradigm akin to the Pyramids and Palm Trees test (Howard and Patterson, 1992). Participants were required to indicate which of the two choice stimuli that appeared at the bottom of the screen was more closely related to the target stimulus shown at the top of the screen. The control condition was a perceptual matching task on scrambled pictures or scrambled words with instructions to select which of the 2 stimuli below was a vertically flipped mirror-image of the target above. This perceptual matching baseline condition controlled for visual processing and task difficulty, but was not informative about how the LATL region of interest contributed to semantic decisions. This leaves open questions about what LATL is actually doing within the semantic system. The current study considers three possible roles for the LATL during semantic matching: (A) recognising object concepts (e.g. the concept ‘grapes’ from a word or picture of ‘grapes’); (B) searching for associations related to each object concept (e.g. ‘wine’ from the presentation of ‘grapes’); and (C) retrieving a semantic concept of interest (e.g. the concept ‘fruit’ that is common to presentations of ‘grapes’ and ‘pears’). See top panel of Fig. 1 for further details.

Other neuroimaging studies of lexical and semantic processing have identified additional anterior temporal lobe areas where activation increases during semantic categorisation (Bright et al., 2004; Devlin et al., 2000**;**Kellenbach et al., 2005 ; Phillips et al., 2002; Mummery et al., 1998; Noppeney and Price 2002a, 2002b; Thierry et al., 2003**;** Thierry and Price, 2006; Vandenberghe et al., 1996; Visser et al., 2010a; Visser and Lambon Ralph 2011). However, the reported locations of these activations varied across studies. Most of the reported effects were more mesial, more posterior or more superior to the LATL region reported in the Pobric et al. (2010) and Visser et al. (2012) studies discussed above. More specifically, the LATL area identified in Pobric et al. (2010) is located just in front of the AC vertical line (*x*=−53, *y*=+4, *z*=−32, in MNI space) and lies in the temporo-polar cortex. In contrast, Vandenberghe et al. (1996) reported more posterior semantic matching activation at [*x*=−44, *y*=−10, *z*=−28]; Bright et al. (2004) reported more medial and posterior semantic matching activation at [*x*=−30, *y*=−18, *z*=−26]; and Thierry and Price (2006) reported more superior semantic matching activation at [*x*=−58, *y*=+8, *z*=−16].

**Fig. 2:**
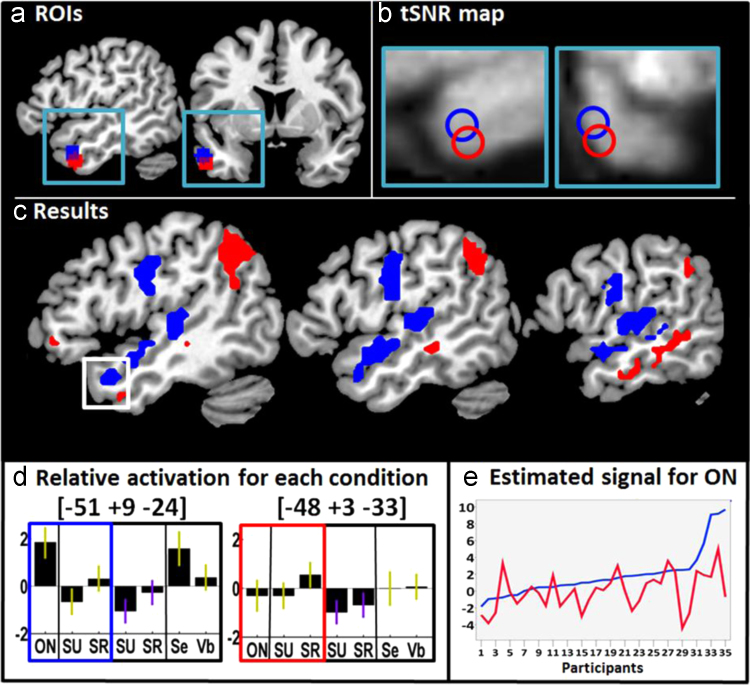
Illustrations of the regions of interest and results. (a) ROI location. The blue ROI is centred on co-ordinates [−54, +6, −26] from Rogers et al. (2006); the red ROI is centred on co-ordinates [−53, +4, −32] from Pobric et al. (2006). Each ROI is a sphere with 6mm radius. (b) The same ROIs are shown as open circles on the temporal signal to noise ratio (tSNR) map to illustrate that our fMRI acquisition protocols produced good LATL signal. (c)Left hemisphere activation for object naming (ON) relative to matching semantically related stimuli (SR), thresholded at p<0.05 uncorrected, after removing activation that is shared by both semantic conditions. Activation shown in blue is for ON>SR. Activation shown in red is the reverse (SR>ON). The location of LATL activation is highlighted within a white square. (d) Relative activation for each condition in each sub-region at the peak co-ordinates from ON>SR in the Rogers sub-region (left) and SR>SU in the Pobric sub-region (right). The height of the bars is the mean effect across subjects in arbitrary units, with 0 corresponding to fixation. Green bars denote visual stimuli, purple bars auditory stimuli. [ON=Object Naming, SU=Semantically Unrelated trials on the Semantic association task, SR=Semantically Related trials on the Semantic association task, Se=Sentence production, Vb=verb naming]. (e) A plot showing how intersubject variability in the effect size for object naming dissociates in the two regions. Blue is the response at the Rogers peak coordinate [−51, +9, −24], red is the response at the Pobric peak coordinate [−48, +3, −33]. Subjects are ordered by the strength of their response in the Rogers region (blue). (For interpretation of the references to color in this figure legend, the reader is referred to the web version of this article.)

From our review of the literature, the lateral ATL area reported in Pobric et al. (2010) at [*x*=−53, *y*=+4, *z*=−32] was closest to that reported by Rogers et al. (2006) at [*x*=−54, *y*=+6, *z*=−26] when healthy participants made category verification decisions on pictures at a specific level (e.g. Is this a picture of a robin?), relative to intermediate (e.g. bird?), or general (e.g. animal?) levels. Without further investigation, however, we do not know if (a) the LATL regions reported in Pobric et al. (2010) and Rogers et al. (2006) are functionally equivalent; or (b) the same region(s) are activated during object naming as well as semantic association and category verification tasks. Therefore, in addition to asking whether LATL activation during semantic matching is related to (A) recognition of the object concepts, (B) search for semantic associations, or (C) retrieval of semantic concepts of interest, we also considered whether LATL activation is related to (D) retrieval of specific concepts as required for naming (See Fig. 1, bottom panel). To accommodate the possibility that the area reported by Pobric et al. (2010) is functionally distinct from the area reported by Rogers et al. (2006), we created 2 different overlapping regions of interest (spheres with 6mm radius, see Fig. 2a), centred on the peak co-ordinates of each area.

Our experimental paradigm included 5 different tasks that systematically manipulated the demands on semantic processing and lexical retrieval. Our first analysis focused on semantic matching and naming responses to pairs of visually presented drawings of objects or animals. During the semantic matching task, the participants were instructed to indicate with a finger press response whether the two objects were either semantically unrelated (SU, e.g. “rabbit and cheese”) or semantically related (SR. e.g. “grape and pear”). During the object naming (ON) task, the objects were always semantically unrelated and participants had to name both objects aloud. According to our task analysis, we assumed that all three conditions (i.e. ON, SU and SR) involve object recognition of each stimulus. Therefore, if LATL contributes to the recognition of the object concepts in the stimuli (hypothesis A), it should be activated, relative to fixation, irrespective of the task or the relationship between the two objects (SR or SU). Second, we assumed that activity involved in making semantic associations should be higher for a task that explicitly requires semantic associations (semantic matching) than a task that is focused on retrieving the verbal representation of the object (i.e. object naming). Therefore, if LATL contributes to semantic search (hypothesis B), it should be more activated by semantic matching (on related or unrelated trials) than object naming. Third, we assumed that only SR trials would involve the successful retrieval of a concept or category that is associated with both stimuli. Therefore, if LATL is related to the retrieval of a semantic concept/category of interest (hypothesis C), then it should show higher activation for SR than SU trials. Fourth, we assumed that the extraction of the stimulus specific concept, as required for naming, should be greater for naming than semantic matching on either SR or SU trials. Therefore, if LATL contributes to retrieval of a specific concept to be named (hypothesis D), then it should be more activated for object naming than semantic matching. For a summary of levels of processing, conditions and contrasts of interest see Table 1.

After investigating the contribution of LATL to semantic processing and object naming, we investigated how LATL activation varied across the other conditions in our paradigm. In our second analysis, we compared semantic matching on heard (i.e. auditory) object names and visually presented pictures to investigate whether semantic processing in LATL was amodal (i.e. independent of stimulus modality). In our third analysis, we compared LATL activation during the production of sentences (e.g. “The horse jumped over the fence”), verbs (e.g. “jumping”) and object names (e.g. “horse and fence”). This allowed us to test whether the semantic matching areas corresponded to areas where activation is enhanced by sentence processing or whether activation was related to the demands on lexical retrieval (more for producing two object names than a single verb name).

## 2 Methods

### 2.1 Participants

Data from 35 healthy, right handed, native English speakers with normal or corrected-to-normal vision were included in this study (mean age: 38.97 years, SD: 15.55). Handedness was assessed with the Edinburgh Handedness Inventory (Oldfield, 1971). The study was approved by the London Queen Square Research Ethics Committee and all subjects gave written informed consent prior to scanning.

### 2.2 Experimental design

The tasks of most interest were semantic matching and object naming. For both tasks, each stimulus presented drawings of two objects. During the object naming task (ON), participants had to name both objects aloud. During the semantic matching task, the participants were instructed to indicate with a finger press response whether the two pictures were either semantically unrelated (SU, e.g. “rabbit and cheese”) or semantically related (SR, e.g. “grape and pear”). Half the stimuli (20 object pairs) were semantically related (SR) and the other half (20 object pairs) were semantically unrelated (SU), see Appendix for details. All semantically related items were associated with one another; 13 also came from the same category (e.g. fruit, vehicle, type of animal, type of tool, musical instrument). The remaining 7 came from a different category (e.g., dolphin–sea, cowboy–horse) but permitted a specific concept of interest (e.g. a category relation: sea creature or syntagmatic relation: cowboys ride horses). For all related object pairs, there may be a number of features that are common to both objects. For example, grapes and pear might be associated at the level of function (edible), colour (green), taste (sweet) and more generally at the category level (fruit), see top panel of Fig. 1. Our assumption was that the retrieval of a concept that links the two items emerges when activity in one or multiple matching features surpasses a threshold for a relatedness decision.

Three other tasks were included: semantic matching on heard object names, sentence production and verb production. Semantic matching on heard object names was the auditory equivalent of the semantic matching task on pictures. Participants listened to two object names and they had to decide if they were either semantically unrelated (SU) or semantically related (SR). Sentence production and verb production were very similar to the object naming condition but instead of presenting drawings of 2 independent objects, the stimuli depicted events of one object interacting with another, for example, a cat drinking from a jug. During the sentence production task, participants had to produce a sentence that linked the two objects with a verb (i.e. “The cat is drinking from the jug”). During the verb production task, participants had to produce the verb only (e.g. “Drinking”). To constrain the number of possible responses, we only used 4 different verbs (i.e. drinking, eating, falling or jumping) in all “events” (sentence and verb production). However, two different objects were presented in each stimulus (with no repetitions across trials or conditions). Consequently, the demands on lexical retrieval were much less during verb naming than sentence production or when 2 independent objects were being named. This provided a measure of how the demands on lexical retrieval influenced LATL activation. Comparison of activation for sentence production to that for verb production and object naming also allowed us to test the influence of sentence processing.

#### 2.2.1 Stimulus selection/creation

Stimulus selection began by generating 60 pictures of easily recognisable animals (e.g. zebra) eating, drinking and jumping in relationship to another object (e.g. a donkey eating a carrot) or objects falling in relation to another object (e.g. a camera falling off a chair). In total, there were 120 animals and objects included in the pictures, all of which had one to four syllable names in English (mean=1.59; SD=0.73). These stimuli were used for the sentence production and verb-naming task.

**Fig. 3:**
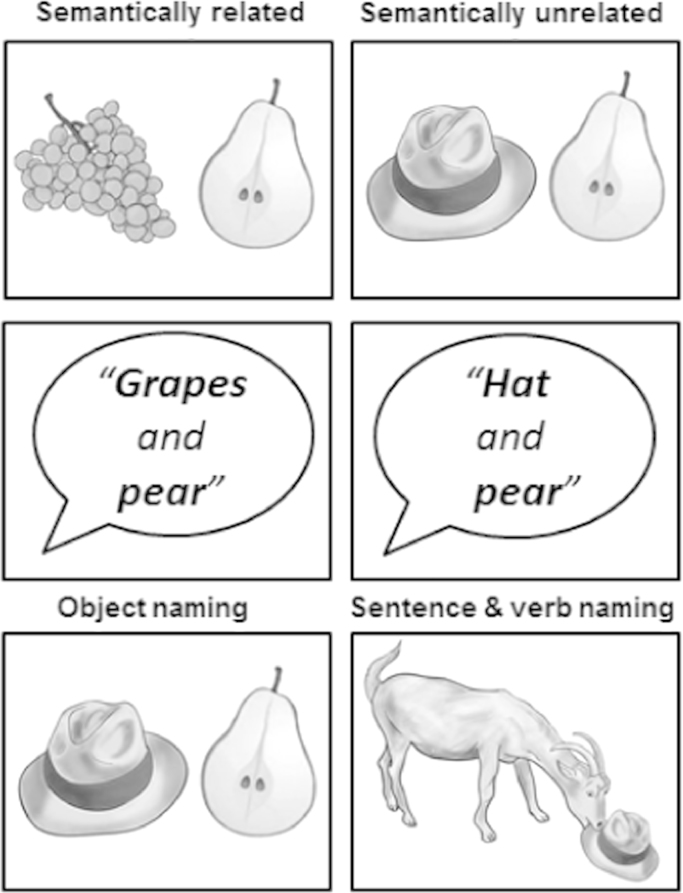
Illustration of stimuli used in the semantic matching and naming tasks. Top row: Examples of semantically related and semantically unrelated stimuli used for semantic matching decisions on pictures of 2 objects. Middle row: In the auditory conditions, object names were spoken in a male voice. Words in a pair were always linked with *“and”*. Bottom row: In the object naming condition, the participant named two objects that were semantically unrelated. In the sentence and verb naming conditions, participants saw pictures of events and either produced the whole sentence (“The goat is eating the hat”) or the verb (“Eating”). Note that the figure shows the same set of four objects (grapes, pear, hat, and goat) in all conditions. However, in the fMRI experiment, participants never saw more than one repetition of the same object, see methods and appendix for further details.

From these 60 event pictures, we generated 120 pictures of individual objects. The stimuli were then paired in novel combinations (i.e. not the same as those used in the events). For example, the stimulus “cup” was presented with “king” in an event picture (“The king is drinking from the cup”) but paired with “teapot” during semantically related matching trials (“cup” and “teapot”) and “piano” during object naming (“cup” and “piano”). Examples of the picture stimuli can be seen in Fig. 3. The stimuli used for the auditory semantic decisions were spoken object names, linked with “and” (e.g. “tiger and bowl”) with a mean duration of 1.92 s and standard deviation of 0.13 s from the onset of the first word to the offset of the second word.

In a pilot study with 26 subjects, the presentation of each of the 120 objects was fully counterbalanced across conditions in different subjects. This allowed us to assign items to conditions that produced the most consistent responses across participants (>80% agreement). For example, items with low name agreement were assigned to the semantic matching conditions. To increase the number of trials, 40/120 objects were repeated once (i.e. occurred in two different conditions) but the pairings of objects was always trial specific. The remaining 80 objects were assigned to one condition only. In post-hoc analyses, we ensured that none of the effects of interest could be explained by a difference between new versus repeated items.

The stimuli and task order were held constant across all 35 participants with the two semantic matching tasks counterbalanced with the three naming tasks, within subjects. The order of conditions for all subjects was: (1) semantic matching on pictures of two objects; (2) naming the objects in pictures; (3) naming the verb in an event (i.e. drinking, eating, falling, or jumping); (4) naming whole sentences; and (5) semantic matching on two heard object names (see Table 2). Related and unrelated semantic matching trials were counterbalanced within task 1 and task 5 (see Table 2 and Appendix for full details). The order of tasks or the stimuli used in different conditions was not counterbalanced across participants because our aim in future studies is to be able to dissociate groups of subjects who vary in their functional anatomy and this post-hoc categorisation requires us to keep the task order and stimuli identical across all individuals. Note that task and stimulus differences, across conditions, cannot explain activation differences for (a) semantically related versus unrelated trials which were counterbalanced within condition with the same number of repetitions; or (b) common effects of relatedness across visual and auditory modalities.

#### 2.2.2 Procedure

Prior to scanning, each subject was familiarised with the tasks using object concepts and stimuli that were not used in the scanner (i.e. different to the 120 objects described above). Each of the 5 tasks was presented in a separate scan run with 4 blocks of 5 object pairs, presented at a rate of one every 5 s (i.e. 10 objects in 25 s blocks). Within any of the semantic matching blocks (visual and auditory), there were 2 or 3 semantically related pairs and 3 or 2 semantically unrelated pairs. Each stimulus block was followed by 16-s resting periods, during which participants fixated on a cross centred on the screen. These resting periods allowed activation to return to baseline between blocks, therefore ensuring maximum sensitivity to all effects of interest. Participants were asked to keep their eyes open for all tasks, including the auditory task, and this was monitored with eye-tracking. They were also frequently reminded to remain as still as possible to prevent movement-induced acquisition noise.

Every stimulus block was preceded by a written instruction (e.g. “Name object”), lasting 3.08 s (equivalent to one interscan interval), which indicated the start of a new block and reminded subjects of the task. All visual stimuli were presented via an LCD projector, and an adjustable head-coil mirror, onto a screen that was clearly visible to the subject (1024×768 resolution). The duration of each visual stimulus was 2.5 s, with 3.5 s fixation between stimuli (i.e. 5 s interstimulus interval). All pictures were scaled to 350×350 pixels and subtended a visual angle of 7.4°.

Auditory stimuli were presented for 1.76–2.5 s via MRI compatible headphones (MR Confon, Magdeburg, Germany), which filtered ambient in-scanner noise. During stimulus presentation, participants fixated on a cross centred on the screen to control for visual input. Volume levels were adjusted for each subject before scanning. Spoken responses were recorded for each subject via a noise-cancelling MRI microphone (FOMRI III^TM^ Optoacoustics, Or-Yehuda, Israel), and transcribed manually for off-line analysis. For auditory and visual semantic decision tasks, participants used two fingers of the same hand (left hand for 15 subjects) to press one of two buttons on an fMRI compatible button box.

### 2.3 Behavioural data processing

A tick sheet with the expected response to each stimulus, in the order of presentation across tasks, enabled us to transcribe responses during the scanning sessions for each participant. Responses were verified by re-listening to the audio-files for each spoken task, and by reviewing the recorded data from the button-press tasks. Each response was categorised as “correct” (i.e. when response matched target) or “incorrect” for all other trials (i.e. when the response did not match the target, was delayed or self-corrected). If >10% of participants produced a spoken response that did not match the target but did match the meaning (e.g. target=mug, response=cup), responses for these participants were also marked as “correct.” In the sentence and object naming conditions, if one of the items was not correct, then the trial was classified as “incorrect”. All participants were more than 80% accurate on all 5 conditions.

Reaction times (RTs) for spoken responses were obtained from the audio files. To compute them, we used an adaptive moving window filter that was tailored to each subject. The optimal window length (i.e. the width which maximally smoothed the audio stream) was based on a portion of the audio file collected during rest. After smoothing the whole time series, we defined the onset of speech as a rise in the absolute amplitude of the smoothed audio stream beyond 1.5 standard deviations from the mean.

### 2.4 MRI acquisition parameters

Functional and structural data were collected using the same acquisition sequences on two 3 T scanners (both made by Siemens, Erlangen, DE) with a 12-channel head coil (20 participants with scanner A and 15 with scanner B). Two sample *t*-tests showed no effects of scanner (*p*>0.05) in either Pobric or Rogers sub-regions for any of the conditions of interest. Total scanning time was approximately 1 h and 30 min per subject, including set up and the acquisition of a structural scan.

Functional images consisted of a gradient-echo EPI sequence (TR/TE=3080 ms/30 ms; Flip angle=90°; matrix size=64×64; FOV=192×192, slice thickness=2 mm, interslice gap=1 mm). Each functional run consisted of 66 volumes per time series, including 5 “dummy scans” to allow for T1 equilibration effects. The TR was chosen to maximise whole-brain coverage (44 slices) and ensure a good coverage of the anterior temporal lobe areas of interest (see Fig. 2b for an illustration of the temporal signal to noise map in the LATL). It also allowed us to asynchronise the slice acquisition with stimulus onset to allow for distributed sampling of stimulus onset across slices in each condition (Veltman et al., 2002).

For anatomical reference, a T1 weighted structural image was acquired after the EPIs, using a 3-D modified driven equilibrium Fourier transform (MDEFT, Deichmann et al., 2004) sequence (TR/TE/TI=7.92 ms/2.48 ms/910 ms, Flip angle=16, 176 slices, voxel size=1×1×1 mm^3^).

### 2.5 fMRI data analysis

#### 2.5.1 Data pre-processing

We performed MRI data analysis in SPM12 (Wellcome Trust Centre for Neuroimaging, London, UK), running on MATLAB 2012a (MathWorks, Natick, MA, USA). Functional volumes were (a) spatially realigned to the first EPI volume and (b) un-warped to compensate for non-linear distortions caused by the interaction between head movement and magnetic field inhomogeneity. The anatomical T1 image was (c) co-registered to the mean EPI image which had been generated during the realignment step and then spatially normalised to the Montreal Neurological Institute (MNI) space using the new unified normalisation-segmentation tool of SPM12. To spatially normalise all EPI scans to MNI space, (d) we applied the deformation field parameters that were obtained during the normalisation of the anatomical T1 image. The original resolution of the different images was maintained during normalisation (voxel size 1×1×1 mm^3^ for structural T1 and 3×3×3 mm^3^ for EPI images). After the normalisation procedure, (e) functional images were spatially smoothed with a 6 mm full-width-half-maximum isotropic Gaussian Kernel to compensate for residual anatomical variability and to permit application of Gaussian random-field theory for statistical inference (Friston et al., 1995).

#### 2.5.2 First level statistical analyses

In the first-level statistical analyses, each pre-processed functional volume was entered into a subject specific, fixed-effect analysis using the general linear model (Friston et al., 1995). All stimulus onset times were modelled as single events, with only the correct response trials as regressors of interest and three extra regressors that included the instructions, incorrect or “other” (self-corrected, delayed or no-response) trials. For semantic matching, different regressors modelled correct trials that were semantically related (SR) or semantically unrelated (SU). For the naming conditions, different regressors modelled new stimuli (not seen in semantic matching) and repeated stimuli (seen in semantic matching). Stimulus functions were then convolved with a canonical hemodynamic response function. To exclude low-frequency confounds, the data were high-pass filtered using a set of discrete cosine basis functions with a cut-off period of 128 s. The 7 contrasts of interest compared each of the different conditions (correct trials only) to fixation. Separate contrasts were generated for semantically related and unrelated trials in the semantic matching tasks (pictures of objects or their heard names). For object naming, the statistical contrasts summed over new and repeated items in the analyses described below because (i) repetition was not a factor of interest, and (ii) there was no significant difference between novel and repeated naming conditions in our regions of interest.

#### 2.5.3 Second level statistical analyses

##### 2.5.3.1 Analysis 1: semantic matching and object naming

A within-subjects one-way ANOVA was performed with three conditions: (1) object naming (ON), (2) semantic matching on related trials (SR) and (3) semantic matching on unrelated trials (SU). The statistical contrasts tested our four hypotheses as follows. Hypothesis A: If LATL activation is related to recognition of the object concepts, then it should be activated for all three conditions relative to fixation. Hypothesis B: If LATL activation is related to semantic association search, then it should be more activated when searching for a semantic association (either SU or SR) than during object naming (ON). Hypothesis C: If LATL activation is related to retrieval of a semantic concept of interest, then it should be more activated when a semantic association common to 2 objects is identified (SR) than not identified (SU). Hypothesis D: If LATL activation is related to retrieval of a specific semantic concept for naming, then it should be more activated for object naming (ON) than semantic matching on either related or unrelated items (SR or SU). These hypotheses were tested with a hierarchical set of statistical comparisons: (1) ON, SR and SU>fixation (inclusively masked with each of the three conditions relative to fixation, (2) SU>ON, (3) SR>SU, (4) ON>SR, see Table 1. The statistical threshold for all the analyses (i.e. Analysis 1, 2 and 3) was *p*<0.05 uncorrected with two small volume searches within the ATL regions of interest (spheres 6 mm radius) centred on the co-ordinates from Pobric et al. (2010) at [−53, +4, −32] and Rogers et al. (2006) at [−54, +6, −26]. The reported *p* Values were corrected for multiple comparisons within each region of interest using the family wise error (FWE).

Finally, an additional analysis was performed to quantify the influence of head motion on our results. We extracted the mean translation length and the mean grade of rotation and used a Pearson Correlation analysis to evaluate whether greater motion was related to the strength of activation during object naming or semantic matching. Motion estimates for object naming and semantic matching were also compared directly using a Paired sample *t*-test.

##### 2.5.3.2 Analysis 2: semantic relatedness on auditory and visual stimuli

The effect of sensory modality (visual or auditory) on semantic relatedness was tested using a 2×2 within subjects factorial design. Factor 1 was semantic relatedness (SR versus SU). Factor 2 was sensory input. The statistical analysis tested for the main effect of relatedness (SR>SU), the main effect of sensory input (auditory versus visual) and the interaction between these factors.

##### 2.5.3.3 Analysis 3: sentence processing and lexical retrieval

A within-subjects one-way ANOVA was performed on the three naming conditions: Sentence production (Se), verb production (Vb) and object naming (ON). Three statistical contrasts compared (1) Se>ON, (2) Se>Vb and (3) ON>Vb. Activation related to sentence processing was expected to show an effect of Se>ON and Se>Vb. Activation related to lexical retrieval was expected to show an effect of Se>Vb and ON>Vb.

### 2.6 Behavioural data analysis

The behavioural data were analysed with SPSS (IBM SPSS, NY, US), with three sets of analyses to compare accuracy and RTs from the conditions involved in the three different fMRI analyses. One-way ANOVAs were used to compare the three conditions in Analysis 1 (i.e. ON, SU and SR) and Analysis 3 (i.e. Se, Vb and ON). A 2×2 repeated-measures ANOVA with relatedness and sensory input as factors was used to compare the conditions included in Analysis 2 (i.e. visual and auditory SU and SR conditions).

## 3 Results

### 3.1 Behavioural results

For summary of the accuracy and RTs for each condition, see Table 3.

#### 3.1.1 Analysis 1: semantic matching and object naming

There were no significant differences in either accuracy or RTs for the three different conditions. This was predicted on the basis of our pilot study that attempted to select items with the most consistently accurate responses. Indeed, accuracy was above 89% for all 20 semantic matching trials and above 80% for each of the 35 participants in all the conditions.

#### 3.1.2 Analysis 2: semantic relatedness on auditory and visual stimuli

There were no significant differences in accuracy for the four different conditions. Accuracy was above 89% for all trials with the exception of one unrelated trial in the auditory condition (“clown and gloves”) that was classed as semantically unrelated by 74% of participants, and semantically related by the remaining 26% of participants. As we mentioned before, only activation related to the correct trials was included in any of the imaging analyses.

Response times were slower for unrelated compared to related conditions (*F* [1, 34]=4.1; *p*=0.05). This is expected given that participants may spend more time searching for semantic associations when there is no obvious semantic link between two objects (in unrelated trials) than when there is a semantic link (in related trials).

Responses were also slower for the auditory compared to visual conditions (*F* [1, 34]=1971.8; *p*<0.05). This was expected because the auditory stimuli were presented sequentially (one object name after another) whereas the visual stimuli presented two pictures of objects simultaneously. Matching semantic associations was therefore delayed relative to stimulus onsets in the auditory modality.

The effect of relatedness (SU>SR) was stronger for auditory stimuli (57 ms) than visual stimuli (27ms) but this interaction did not reach significance (*F* [1, 34]=0.96 *p*>0.05).

#### 3.1.3 Analysis 3: sentence processing and lexical retrieval

Across conditions, there were significant differences in accuracy (*F* [2,102] =11.6; *p*<0.001) and RTs (*F* [2,102]=16.1; *p*<0.001). A set of post-hoct-tests (Fisher’s Least Significant Difference tests), showed that these differences were due to higher accuracy in Vb relative to Se and ON conditions, and slower responses for Se relative to Vb and ON conditions (all *p*<0.001 after Bonferroni corrections for multiple comparisons).

### 3.2 Neuroimaging results LATL

#### 3.2.1 Analysis 1: semantic matching and object naming

A. *Object recognition*. LATL was not activated, in either subregion, for all 3 tasks (ON, SR, SU) relative to fixation.
B. *Search for semantic associations*. LATL was not activated, in either subregion, for semantic matching on unrelated items relative to object naming.
C. *Retrieval of an associated concept*. In both LATL subregions, activation was greater for matching semantically related objects (e.g. tree and log) than semantically unrelated objects (e.g. tree and pillow). This suggests both areas are involved in the successful retrieval of concepts of interest (Semantic Retrieval). See Table 4 and column 2 and 3 in the plots of Fig. 2d.
D. *Retrieving the stimulus specific concept for naming*. In the Rogers et al. (2006) subregion, activation was higher for ON than SR (Table 4 and the first 3 columns of the plots in Fig. 2d). This was not the case in the Pobric sub-regions, where we found a trend (*Z*=2.0) for a reverse effect (SR>ON).

To test for the region by condition interaction, we conducted a two-way ANOVA in SPSS with conditions (ON and SR) and sub-regions (Rogers and Pobric areas) as factors. The data entered were extracted from the first eigenvariate in SPM, at the peak co-ordinates of our effects of interest (ON>SR and SR>SU). As expected from the SPM results, the effect of sub-region (*F* [1, 34]=8.30; *p*<0.01) interacted with the effect of condition (*F* [1, 34]=15.68; *p*<0.001). The statistics related to the post-hoc tests showed significantly more activation for ON than SR in the Rogers subregion (*T* (34)=2.86; *p*<0.01), and for ON in the Rogers subregion compared to the Pobric region (*T* (34)=4.07; *p*<0.001). Further evidence that the functional responses dissociated in our two regions of interest became apparent when we compared the pattern of inter-subject variability for ON in each region. As shown in Fig. 2e, there was no co-dependency between regions, despite their proximity. This suggests that the regions were being driven by different types of processing.

##### 3.2.1.1 Additional analysis

Finally, we note that the functional dissociation between our two regions of interest cannot be explained by motion artefacts because there was no significant correlation between any movement parameter and BOLD response in either of our regions of interest (*R* ranges from −0.2 to 0.2, *p* Values ranges from 0.2 to 1). Thus although mean translation length and mean grades of rotation were greater during naming [1.06 mm (±0.5) and 0.39° (±0.2)] than semantic matching [0.56 mm (±0.4) and 0.19° (±0.1)]; with these differences reaching significance [*t* (34) =−7.01, and −7.12, *p*<0.001], this effect did not impact upon one region more than another.

#### 3.2.2 Analysis 2: semantic relatedness on auditory and visual stimuli

In both subregions, there was a main effect of related versus unrelated semantic decisions (across visual and auditory modalities). In the more inferior region, identified by Pobric et al. (2010), there was also a main effect of stimulus modality (visual>auditory across relatedness), with a corresponding but non-significant trend in the area identified by Rogers et al. (2006). The effects of stimulus modality and semantic relatedness were additive with no significant interaction (see Table 4 and columns 2 to 5 in Fig. 2d). This confirmed that the effect of semantic relatedness was observed in both modalities. The *Z* scores for SR>SU in the auditory modality only were 3.42 in the Rogers sub-region and 2.47 in the Pobric sub-region.

#### 3.2.3 Analysis 3: sentence processing and lexical retrieval

In the Rogers et al. (2006) subregion that was more activated by naming than semantic matching (ON>SR in Analysis 1), activation was higher for sentence naming and object naming relative to verb naming (consistent with the demands on lexical retrieval), but did not differ for sentence naming and object naming (see Table 4 and columns 1, 6 and 7 in the Fig. 2d). In the Pobric et al. (2010) subregion that was more activated for matching semantically related items than naming (SR>ON in Analysis 1), there was no significant effect of either sentence production or lexical retrieval (see Table 4 and column 1, 6 and 7 in the plots of Fig. 2d).

## 4 Discussion

Our investigation into the role of LATL in semantic processing unexpectedly revealed different functional responses in the subregion identified by Pobric et al. (2010) and the subregion identified by Rogers et al. (2006). Both regions were more activated when participants identified a common semantic association for two objects (SR>SU). In addition the Rogers et al. (2006) region was most activated by object naming and the demands on lexical retrieval.

Our finding that the Rogers et al. (2006) area is related to the retrieval of a semantic concept of interest (SR>SU) is consistent with prior conclusions, but here we additionally show for the first time how its activation during the retrieval of specific semantic information is increased in object naming (ON>SR), and with lexical retrieval demands (ON>Ve). Likewise, our finding that the Pobric et al. (2010) area is activated by semantic matching is also consistent with prior conclusions but here we additionally show that the effect is being driven by the retrieval of a semantic concept of interest during semantically related rather than unrelated trials.

### 4.1 Neurocognitive models of semantic memory

Our finding that both LATL sub-regions were more activated for semantically related than unrelated trials during the semantic matching task illustrates that both our LATL subregions are involved in retrieving abstract conceptual information that is over and above the individual inputs. This is consistent with neurocognitive models that describe the ATL as a semantic integration area (or hub) where modality-specific information from distributed brain areas (the spokes) is linked into the amodal semantic similarity structure that underpins semantic concepts or representations (Binney et al., 2012; Patterson et al., 2007; Lambon Ralph and Patterson, 2008; Lambon Ralph, 2013; Rogers et al., 2004a). More specifically, higher activation for retrieving conceptual information when making semantic matching decisions is consistent with the extra layer of processing proposed in the semantic hub account which enables the semantic system to extract new concepts and generalise across different exemplars of the same concept based on semantic features rather than modality-specific characteristics (Lambon Ralph et al., 2010; Rogers et al., 2004a). For example, in our task, different stimuli (e. g. pears and grapes) could be matched based on their membership of a shared category (e.g. fruit) rather than on their modality specific features of colour, taste, or shape.

A second relevant finding was that, in both LATL subregions, activation for semantically related compared to unrelated trials was observed for auditory inputs (heard object names) as well as visual inputs (pictures of objects). This is consistent with the amodal nature of the semantic system that was initially proposed on the basis of studies of patients with extensive ATL damage who have difficulties with semantic processing irrespective of the sensory modality of the input (Bozeat et al., 2000; Coccia et al., 2004; Goll et al., 2009; Lambon Ralph et al., 2001; Piwnica–Worms et al., 2010, Rogers et al., 2004b). Although functional imaging studies have reported ATL activation related to semantic processing of auditory words and sounds (Noppeney and Price, 2002a, 2002b; Thierry and Price, 2006; Visser and Lambon Ralph 2011), the location of effects varied markedly across studies and did not overlap with the LATL areas that are the focus of the current study. Plausibly the lack of reported LATL activation in previous studies of both visual and auditory semantics could be a consequence of (a) the LATL subregions being part of a large area of inferior temporal activation where the peak effect is more posterior to our areas of interest although it extended into our regions of interest (e.g. Visser and Lambon-Ralph, 2011); (b) reports of effects that sum over semantically related and unrelated trials; (c) exclusion of the inferior temporal lobe during data acquisition; or (d) insensitivity to inferior temporal lobe activation due to fMRI susceptibility artifacts (Devlin et al., 2000; Visser et al., 2010b). In sum, our finding of LATL activation that was common to visual and auditory semantic processing is consistent with the semantic hub account which posits that the semantic content of all types of stimuli are processed irrespective of the sensory modality that they are presented in. This creates modality-invariant concepts that enable the cross-modal translation between the modality specific sources of information.

A third novel finding was the main effect of sensory input (pictures of objects>heard object names) in the more inferior LATL subregion identified by Pobric et al. (2010). This was additive with the effect of semantic relatedness (related>unrelated). We speculate that this occurred because pictures have multiple semantic associations that may need to be resolved to identify a concept of interest whereas words may initially invoke more specific concepts and restrict search. In our design, it was also the case that the duration of the picture stimuli (2.5 s) was longer than the duration of the auditory words because 2 objects were presented simultaneously in pictures and stayed on the screen for 2.5 s; however auditory stimuli were presented sequentially. Retrieval of a semantic concept of interest will therefore be sustained for longer during the picture condition than the auditory word condition.

A previous meta-analysis (Visser et al., 2010b) of semantic tasks (from passive listening to words and sounds, reading, semantic judgment, naming, property verification, lexical decision and verbal fluency) for picture, visual words and auditory words stimuli, suggested a superior–inferior gradation of activation, so that peak activations tended to be more superior for verbal tasks, and more inferior for the picture tasks. This graded contribution of the ATL for auditory and visual semantics was not replicated in our within-subject design (Analysis 2). Instead, as noted above, we found an additive effect of semantic relatedness and stimulus modality (visual>auditory). We therefore speculate that the previously observed differences in the peak semantic activation for auditory and visual semantics are the consequence of confounds from (a) auditory perceptual processing in neighbouring superior temporal areas and (b) richer semantic processing that might be evoked by pictures of objects compared to their spoken names in inferior temporal regions.

### 4.2 Limitations

In this paper, we focused on the functional responses in two left LATL subregions. Many other anterior temporal areas have been associated with language tasks and these areas may have functions that are very distinct from those we report in the current paper. For example, although we did not find any evidence that our LATL sub-regions were sensitive to the demands on sentence production relative to object naming, other superior and middle temporal areas are expected to be selective to sentence comprehension or production (Brennan and Pylkkänen, 2012; Friederici and Kotz, 2003; Friederici and Gierhan, 2013; Grodzinsky and Friederici, 2006; Humphries et al., 2005, 2006; Kaan and Swaab, 2002; Noppeney and Price, 2004; Vandenberghe et al., 2002).

## 5 Conclusions

In this fMRI study, we show the specific contribution of two neighbouring LATL subregions to semantic decisions and object naming. Both are involved in retrieving a semantic concept that is common to two different objects. In addition, we found that the subregion that Rogers et al. (2006) associated with category verification at a specific level (e.g. Labrador) relative to intermediate level (e.g. dog) or general level (e.g. animal) was more activated for object naming than semantic matching and it was also sensitive to the demands on lexical retrieval (Analysis 3). With respect to cognitive models of semantic processing, our functional imaging results illustrate activation consistent with (1) an extra layer of processing when a concept of interest is retrieved; (2) amodal semantic representations; and (3) enhanced semantic associations for picture stimuli relative to auditory object names.

## Conflict of interest

None declared.

## Figures and Tables

**Table A1:** Object names presented in all conditions.

**Stimulus**	**Pictures of objects**	**Pictures of objects**	**Pictures of events**	**Pictures of events**	**Auditory words**
**Task**	**Semantic match**	**Name objects**	**Name action (verb)**	**Produce sentence**	**Semantic match**

**Block 1**	frog_plate	deer_tankard	guitar_falling_from_sofa	boy_jumping_on_bed	“dolphin and sea“
car_truck	fox_boat	kangaroo_jumping_over_car	nurse_drinking_from_glass	“bag and suitcase“
king_stool	zebra_fence	koala_eating_pear	parrot_drinking_from_tap	“penguin and hand“
piano_guitar	goat_train	swan_eating_grapes	pig_eating_pizza	“mermaid and rock“
cup_teapot	lion_man	piano_falling_from_truck	owl_eating_tomato	“clown and gloves“
**Block 2**	cowboy_horse	pizza_tap	potato_falling_from_sack	gloves_falling_from_bag	“chair and leaves“
pirate_boat	baby_kettle	snake_eating_apple	crab_falling_from_hand	“fountain and waterfall“
man_fox	bottle_boy	duck_eating_cake	jacket_falling_from_suitcase	“bird and nest“
train_bus	pig_mug	monkey_eating_a_banana	puppy_eating_shoe	“rabbit and cheese“
hat_swimmingpool	nurse_tomato	pilot_drinking_from_coconut	mermaid_drinking_from_shell	“mouse and cat“
**Block 3**	tankard_girl	hedge_stove	bread_falling_from_basket	clown_jumping_on_trampoline	“elephant and giraffe“
zebra_lion	bed_cow	donkey_eating_carrot	dolphin_jumping_through_hoop	“fridge and camera“
fence_gate	sheep_parrot	nun_falling_from_plane	penguin_eating_fish	“tent and jug“
goat_swing	lamb_glass	book_falling_from_shelf	seal_jumping_from_rocks	“tiger and bowl“
deer_barrel	owl_saucepan	lizard_eating_biscuit	whale_jumping_from_sea	“log and tree“
**Block 4**	grapes_pear	bus_horse	frog_jumping_from_stool	lamb_drinking_from_bottle	“jacket and puppy“
swan_wineglass	swing_cowboy	king_drinking_from_cup	cow_eating_hedge	“shoe and hoop“
tortoise_table	hat_gate	tortoise_drinking_from_plate	baby_drinking_from_mug	“fish and whale“
kangaroo_koala	pirate_swimmingpool	teapot_falling_off_table	sheep_drinking_from_saucepan	“trampoline and seal“
soldier_sofa	girl_barrel	soldier_drinking_from_wineglass	kettle_falling_from_stove	“crab and shell“

**Table 1:** Main levels of processing, conditions and contrasts.

**Levels of processing**	**ON**	**SU**	**SR**	**Contrasts**
**A)** Recognition of the object concepts	✔	✔	✔	All>fixation
**B)** Search for semantic associations	**x**	✔	✔	SU>ON
**C)** Retrieval of a semantic concept for matching	**x**	**x**	✔	SR>SU
**D)** Retrieval of a specific concept for naming	✔	**x**	**x**	ON>SR

ON=Object Naming, SU=Semantically Unrelated trials on the Semantic association task, SR=Semantically Related trials on the Semantic association task, F=fixation.

**Table 2:** Task order and description.

**Task order**	**Stimuli**	**Task instructions**	**Response**
1. Visual sematic matching	2 pictures	Decide if stimuli are related or unrelated	Finger
2. Object naming	2 pictures	Name two unrelated objects/animals	Speech
3. Verb naming	Event picture	Name the verb corresponding to the action	Speech
4. Sentence production	Event picture	Describe in one sentence the action	Speech
5. Auditory semantic matching	2 auditory words	Decide if stimuli are related or unrelated	Finger

Responses were either finger press or overt speech.

**Table 3:** Accuracy [in %] and Response times [in ms] for correct trials (with standard deviation) in each condition.

		**Accuracy**	**RTs**
**Naming**	**ON**	94(5.5)	1330(167)
**Visual**	**SU**	96(6.0)	1271(233)
**Semantics**	**SR**	97(6.7)	1244(232)
**Auditory**	**SU**	95(6.2)	2590(209)
**Semantics**	**SR**	95(6.1)	2533(195)
**Sentences**	**Se**	93(6.0)	1501(219)
**Processing**	**Vb**	98(3.0)	1233(202)

ON=Object Naming, SU=Semantically Unrelated trials on the Semantic association task, SR=Semantically Related trials on the Semantic association task, Se=Sentence production, Vb=verb naming

**Table 4:** Summary of the results as obtained in both LATL sub-regions.

***Levels of processing***	***More inferior sub-region* (****Pobric et al., 2010****)**			***Less inferior sub-region* (****Rogers et al., 2006****)**		
	*MNI*	*Zscore*	*P*_*FWE*_	*MNI*	*Zscore*	*P*_*FWE*_

***Semantic matching and object naming***						
***A)** Object recognition*	*NS*			*NS*		
***B)** Search for semantic associations*	*NS*			*NS*		
***C)** Retrieval of a semantic concept of interest*	*−48+3−33*	*2.8*	*0.024*	*−51+9−24*	*3.0*	*0.025*
***D)** Retrieval of stimulus specific concept*	*NS*			*−51+9*−*24*	*3.2*	*0.011*

***Visual and auditory semantics***						
*Main effect of relatedness*	*−48+3−33*	*3.1*	*0.008*	*−51+9−27*	*4.2*	*0.001*
*Main effect of input modality*	*−51+3−33*	*3.6*	*0.002*	*−54+6−24*	*2.4*	*0.158*
*Interaction*	*NS*			*NS*		

***Sentence and lexical processing***						
*Sentences>verb*	*NS*			*−51+9−24*	*3.1*	*0.016*
*Sentences>ON*	*NS*			*NS*		
*ON>verbs*	*NS*			*−51+9−24*	*3.9*	*0.004*

NS=Not significant.

## Appendix

See Table A1.

## References

[bib1] Brennan J., Pylkkänen L. (2012). The time-course and spatial distribution of brain activity associated with sentence processing. Neuroimage.

[bib2] Binder J.R., Desai R.H., Graves W.W., Conant L.L. (2009). Where is the semantic system? A critical review and meta-analysis of 20 functional neuroimaging studies. Cereb. Cortex.

[bib3] Binder J.R., Desai R.H. (2011). The neurobiology of semantic memory. Trends Cogn. Sci..

[bib4] Binney R.J., Embleton K.V., Jefferies E., Parker G.J., Lambon Ralph M.A. (2010). The ventral and inferolateral aspects of the anterior temporal lobe are crucial in semantic memory: evidence from a novel direct comparison of distortion-corrected fMRI, rTMS, and semantic dementia. Cereb. Cortex.

[bib5] Binney R.J., Parker G.J., Lambon Ralph M.A. (2012). Convergent connectivity and graded specialization in the rostral human temporal lobe as revealed by diffusion-weighted imaging probabilistic tractography. J. Cogn. Neurosci..

[bib6] Bozeat S., Lambon Ralph M.A., Patterson K., Garrard P., Hodges J.R. (2000). Non-verbal semantic impairment in semantic dementia. Neuropsychologia.

[bib7] Bright P., Moss H., Tyler L.K. (2004). Unitary vs multiple semantics: PET studies of word and picture processing. Brain Lang..

[bib8] Coccia M., Bartolini M., Luzzi S., Provinciali L., Lambon Ralph M.A. (2004). Semantic memory is an amodal, dynamic system: evidence from the interaction of naming and object use in semantic dementia. Cogn. Neuropsychol..

[bib9] Deichmann R., Schwarzbauer C., Turner R. (2004). Optimisation of the 3D MDEFT sequence for anatomical brain imaging: technical implications at 1.5 and 3T. Neuroimage.

[bib10] Devlin J.T., Russell R.P., Davis M.H., Price C.J., Wilson J., Moss H.E., Matthews P.M., Tyler L.K. (2000). Susceptibility-induced loss of signal: comparing PET and fMRI on a semantic task. Neuroimage.

[bib11] Friederici A.D., Kotz S.A. (2003). The brain basis of syntactic processes: Functional imaging and lesion studies. Neuroimage.

[bib12] Friederici A.D., Gierhan S.M.E. (2013). The language network. Curr. Opin. Neurobiol..

[bib13] Friston K.J., Holmes A.P., Worsley K.J., Poline J.P., Frith C.D., Frackowiak R.S.J. (1995). Statistical parametric maps in functional imaging: a general linear approach. Hum. Brain Mapp..

[bib14] Goll J.C., Crutch S.J., Loo J.H.Y., Rohrer J.D., Frost C., Bamiou D.-E., Warren J.D. (2009). Non-verbal sound processing in the primary progressive aphasias. Brain.

[bib15] Grodzinsky Y., Friederici A.D. (2006). Neuroimaging of syntax and syntactic processing. Curr. Opin. Neurobiol..

[bib16] Howard D., Patterson K. (1992). Pyramids and palm trees: a test of semantic access from pictures and words.

[bib17] Humphries C., Binder J.R., Medler D.A., Liebenthal E. (2006). Syntactic and semantic modulation of neural activity during auditory sentence comprehension. J. Cogn. Neurosci..

[bib18] Humphries C., Love T., Swinney D., Hickok G. (2005). Response of anterior temporal cortex to syntactic and prosodic manipulations during sentence processing. Hum. Brain Mapp..

[bib19] Kaan E., Swaab T.Y. (2002). The brain circuitry of syntactic comprehension. Trends in Cogn. Sci..

[bib20] Kellenbach M.L., Hovius M., Patterson K. (2005). A pet study of visual and semantic knowledge about objects. Cortex.

[bib21] Lambon Ralph M.A., McClelland J.L., Patterson K., Galton C.J., Hodges J.R. (2001). No right to speak? The relationship between object naming and semantic impairment: neuropsychological evidence and a computational model. J. Cogn. Neurosci..

[bib22] Lambon Ralph M.A., Patterson K. (2008). Generalization and differentiation in semantic memory. Ann. N. Y. Acad. Sci..

[bib23] Lambon Ralph M.A., Pobric G., Jefferies E. (2009). Conceptual knowledge is underpinned by the temporal pole bilaterally: convergent evidence from rTMS. Cereb. Cortex.

[bib24] Lambon Ralph M.A., Sage K., Jones R.W., Mayberry E.J. (2010). Coherent concepts are computed in the anterior temporal lobes. Proc. Natl. Acad. Sci. USA.

[bib25] Lambon Ralph M.A. (2013). Neurocognitive insights on conceptual knowledge and its breakdown. Philos. Trans. R. Soc. Lond. B Biol. Sci..

[bib26] Mummery C.J., Patterson K., Hodges J.R., Price C.J. (1998). Functional neuroanatomy of the semantic system: divisible by what?. J. Cogn. Neurosci..

[bib27] Noppeney U., Price C.J. (2002). Retrieval of visual, auditory, and abstract semantics. Neuroimage.

[bib28] Noppeney U., Price C.J. (2002). A PET study of stimulus- and task-induced semantic processing. Neuroimage.

[bib29] Noppeney U., Price C.J. (2004). An fMRI study of syntactic adaptation. J. Cogn. Neurosci..

[bib30] Oldfield R.C. (1971). The assessment and analysis of handedness: the Edinburgh inventory. Neuropsychologia.

[bib31] Patterson K., Nestor P.J., Rogers T.T. (2007). Where do you know what you know? The representation of semantic knowledge in the human brain. Nat. Rev. Neurosci..

[bib32] Phillips J.A., Noppeney U., Humphreys G.W., Price C.J. (2002). Can segregation within the semantic system account for category-specific deficits?. Brain.

[bib33] Piwnica-Worms K.E., Omar R., Hailstone J.C., Warren J.D. (2010). Flavour processing in semantic dementia. Cortex.

[bib34] Pobric G., Jefferies E., Lambon Ralph M.A. (2007). Anterior temporal lobes mediate semantic representation: mimicking semantic dementia by using rTMS in normal participants. Proc. Natl. Acad. Sci. USA.

[bib35] Pobric G., Jefferies E., Lambon Ralph M.A. (2010). Amodal semantic representations depend on both anterior temporal lobes: evidence from repetitive transcranial magnetic stimulation. Neuropsychologia.

[bib36] Price C.J. (2012). A review and synthesis of the first 20 years of PET and fMRI studies of heard speech, spoken language and reading. Neuroimage.

[bib37] Rogers T.T., Hocking J., Noppeney U., Mechelli A., Gorno-Tempini M.L., Patterson K., Price C.J. (2006). Anterior temporal cortex and semantic memory: reconciling findings from neuropsychology and functional imaging. Cogn. Affect. Behav. Neurosci..

[bib38] Rogers T.T., Lambon Ralph M.A., Garrard P., Bozeat S., McClelland J.L., Hodges J.R., Patterson K. (2004). Structure and deterioration of semantic memory: a neuropsychological and computational investigation. Psychol. Rev..

[bib39] Rogers T.T., Lambon Ralph M.A., Hodges J.R., Patterson K. (2004). Natural selection: the impact of semantic impairment on lexical and object decision. Cogn. Neuropsychol..

[bib40] Thierry G., Giraud A.L., Price C. (2003). Hemispheric dissociation in access to the human semantic system. Neuron.

[bib41] Thierry G., Price C.J. (2006). Dissociating verbal and nonverbal conceptual processing in the human brain. J. Cogn. Neurosci..

[bib42] Vandenberghe R., Nobre A.C., Price C.J. (2002). The response of left temporal cortex to sentences. J. Cogn. Neurosci..

[bib43] Vandenberghe R., Price C., Wise R., Josephs O., Frackowiak R.S. (1996). Functional anatomy of a common semantic system for words and pictures. Nature.

[bib44] Veltman D.J., Mechelli A., Friston K.J., Price C.J. (2002). The importance of distributed sampling in blocked functional magnetic resonance imaging designs. Neuroimage.

[bib45] Visser M., Embleton K.V., Jefferies E., Parker G.J., Lambon Ralph M.A. (2010). The inferior, anterior temporal lobes and semantic memory clarified: novel evidence from distortion-corrected fMRI. Neuropsychologia.

[bib46] Visser M., Jefferies E., Embleton K.V., Lambon Ralph M.A. (2012). Both the middle temporal gyrus and the ventral anterior temporal area are crucial for multimodal semanticprocessing: distortion-corrected fMRI evidence for a double gradient of information convergence in the temporallobes. J. Cogn. Neurosci..

[bib47] Visser M., Jefferies E., Lambon Ralph M.A. (2010). Semantic processing in the anterior temporal lobes: a meta-analysis of the functional neuroimaging literature. J. Cogn. Neurosci..

[bib48] Visser M., Lambon Ralph M.A. (2011). Differential contributions of bilateral ventral anterior temporal lobe and left anterior superior temporal gyrus to semantic processes. J. Cogn. Neurosci..

[bib49] Wong C., Gallate J. (2012). The function of the anterior temporal lobe: a review of the empirical evidence. Brain Res..

